# Oligo- and Polymetastatic Progression in Lung Metastasis(es) Patients Is Associated with Specific MicroRNAs

**DOI:** 10.1371/journal.pone.0050141

**Published:** 2012-12-10

**Authors:** Yves A. Lussier, Nikolai N. Khodarev, Kelly Regan, Kimberly Corbin, Haiquan Li, Sabha Ganai, Sajid A. Khan, Jennifer Gnerlich, Thomas E. Darga, Hanli Fan, Oleksiy Karpenko, Philip B. Paty, Mitchell C. Posner, Steven J. Chmura, Samuel Hellman, Mark K. Ferguson, Ralph R. Weichselbaum

**Affiliations:** 1 Comprehensive Cancer Center, The University of Chicago, Chicago, Illinois, United States of America; 2 Center for Biomedical Informatics, Dept. of Medicine, The University of Chicago, Chicago, Illinois, United States of America; 3 Ludwig Center for Metastasis Research, The University of Chicago, Chicago, Illinois, United States of America; 4 Department of Medicine, University of Illinois at Chicago, Chicago, Illinois, United States of America; 5 Department of Bioengineering, University of Illinois at Chicago, Chicago, Illinois, United States of America; 6 Center for Interventional Health Informatics, University of Illinois at Chicago, Chicago, Illinois, United States of America; 7 Cancer Center, University of Illinois, Chicago, Illinois, United States of America; 8 Dept. of Radiation and Cellular Oncology, The University of Chicago, Chicago, Illinois, United States of America; 9 Department of Surgery, The University of Chicago, Chicago, Illinois, United States of America; 10 Dept. of Surgery, Memorial Sloan-Kettering Cancer Center, New York, New York, United States of America; University of Vermont, United States of America

## Abstract

**Rationale:**

Strategies to stage and treat cancer rely on a presumption of either localized or widespread metastatic disease. An intermediate state of metastasis termed oligometastasis(es) characterized by limited progression has been proposed. Oligometastases are amenable to treatment by surgical resection or radiotherapy.

**Methods:**

We analyzed microRNA expression patterns from lung metastasis samples of patients with ≤5 initial metastases resected with curative intent.

**Results:**

Patients were stratified into subgroups based on their rate of metastatic progression. We prioritized microRNAs between patients with the highest and lowest rates of recurrence. We designated these as high rate of progression (HRP) and low rate of progression (LRP); the latter group included patients with no recurrences. The prioritized microRNAs distinguished HRP from LRP and were associated with rate of metastatic progression and survival in an independent validation dataset.

**Conclusion:**

Oligo- and poly- metastasis are distinct entities at the clinical and molecular level.

## Introduction

Metastases are primary determinant of cancer-related death [1]. The presence of distant metastases in many solid tumors has been synonymous with a fatal outcome with rare exceptions, e.g. chemotherapy for testicular cancer [2], [3]. However, it is increasingly recognized that distant metastases may not always be numerous and widespread. A clinically-limited number of metastases has been designated as “oligometastases” [4], [5]. This hypothesis suggests that a proportion of cells have limited potential for dissemination [6], [7]. Clinical evidence supports the idea that some oligometastases are curable. Surgical series demonstrate long-term survival among patients undergoing resection for liver metastases from gastrointestinal primary tumors colorectal cancer as well as pulmonary metastasectomy for diverse types of tumors [8]–[12]. In addition, stereotactic body radiotherapy (SBRT) has been used to treat limited metastases with favorable long-term survival [13]–[16].

Clinical criteria are used to select patients with limited number of metastases for the local therapies of curative intent [8], [12], [17], [18]. Despite the selection criteria, survival rates of approximately 25% demonstrate that the majority of patients selected for aggressive local metastasis therapy are not cured. A method for accurate classification of oligo- and poly- metastatic patients could have important clinical implications in both assignment of therapy (*e.g.* local vs. systemic) and prognostic value.

MicroRNAs are small, non-encoding RNA molecules that regulate as many as 200 genes. Their expression profiles appear to classify cancers [19]. Several reports suggest that microRNAs better classify cancer subtypes compared to expression profiling of protein coding genes [20], [21]. We previously studied those microRNAs that characterized a small heterogeneous group of primary and metastatic tumor samples in patients with clinically limited metastases [22]. Here, we study a larger, more homogeneous group of patients with resected lung metastasis, and compare our findings to the previous report. Our results indicate that the rate of metastatic progression following resection of patients presenting ≤5 metastases predicts clinical outcome. We identified a prioritized list of microRNAs that accurately reflects the rate of metastatic progression and or metastatic colonization in patients with lung metastasis. These data represent the only known datasets of microRNAs associated with oligometastases.

## Materials and Methods

### I. Clinical data and patient classification of lung metastatic progression

Patients at our institution undergoing lung resection for metastases, consisting of wedge resection, lobectomy, or pneumonectomy, were analyzed. Inclusion criteria for this study required: (i) that patients had 1 to 5 metastasis(es) at first metastatic presentation and had discrete metastasis amenable to therapy (*e.g.* no clinical or radiologic evidence of metastases in the pleural, peritoneal, pericardial or retroperitoneal cavities), (ii) that each patient had at least one lung metastasis resected with formalin fixed, paraffin embedded (FFPE) tissue from the resected lung metastases available for analysis from which the quality control measures of microRNA expression were sufficient for qPCR, and (iii) that at the time of lung surgery, every site of known metastases was treated with definitive intent. For survival analyses, a minimum of 16 months of follow-up after surgery or death at any follow-up period was required. Electronic medical records and imaging were reviewed for clinical parameters of interest, including the number and dates of additional metastasis development. Patient survival was calculated from the time of lung metastasis surgery until death from any cause and living patients were censored at last follow-up. Sixty-three patients meeting clinical criteria with pathology available were identified. This study was conducted with approval on March 16th 2012 from the University of Chicago Institutional Review Board (IRB; Amendment #4 for protocol #17018B - revised protocol v 02.24.12: “Signed Consent Waived”).

**Figure 1:**
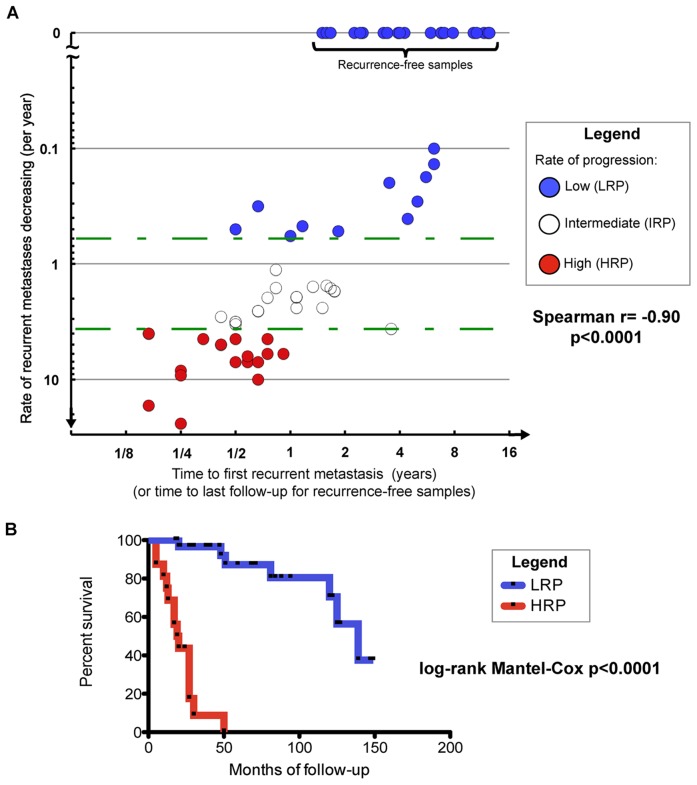
Stratification of metastatic phenotypes in lung metastasis patient samples reveals three distinct subgroups and a difference in survival outcome between patients with high vs. low rates of progression. **A**, lung metastasis patients were stratified according to the time (years) to first recurrent metastasis following pulmonary metastectomy (x-axis) and rate of recurrent metastases (per year) over follow-up after surgery (y-axis). Three distinct subgroups emerged using cutoffs for the rate of recurrent metastases: patients with a high rate of progression develop at least 3.6 new tumors/year after surgery and tend to exhibit their first recurrence in the first year following surgery (HRP, red circles); patients with a low rate of progression develop less than 0.6 new tumors/year after surgery (LRP, blue circles); remaining patients that do not meet the criteria for high or low rate of progression status are classified with an intermediate rate of progression (IRP, white circles). Green dotted lines represent the aforementioned rate thresholds. LRP patients exhibiting no recurrence after metastectomy were also plotted here for convenience as yielding 0.0 metastases/month on the y-axis. See **Tables S1** and **S2** for further clinical information. **B**, lung metastasis patients of the LRP (n = 32) and HRP (n = 16) subgroups were compared for their survival outcome using log-rank Mantel-Cox analysis. The median survival of LRP and HRP patients is 63.5 months and 18 months, respectively. A log-rank Mantel-Cox p<0.0001 was obtained when comparing LRP vs. HRP survival outcome.

The rate of new metastases over the follow-up period after pulmonary metastectomy was plotted against time from surgery to the first metastatic recurrence (**Figure 1**). Three distinct groups were identified: (i) patients with no and/or low rate of recurrence (**LRP**), (ii) patients with a high rate of recurrence (**HRP**), and (iii) patients with an intermediate rate of recurrence (**IRP**). In order to evenly distribute patients with metastatic recurrence between these three groups, we selected rate thresholds of <0.6 new metastases per year (including those patients with no evidence of disease), of >3.6 new metastases per year, and of 0.6 to 3.6 metastases per year, respectively. To enhance the signal between groups, we focused our comparison on the two diametric extreme groups: HRP and LRP patients. Further, in order to compare these metastatic phenotypes with our prior published results [22], we also classified patients by poly- (**PM**) vs. oligo-metastatic (**OM**) progression. PM was defined within ≤18 months following first metastatic recurrence as either (i) developing >5 new recurrent metastases in a timespan ≤4 months or (ii) progression within a body cavity (i.e. pericardial, pleural, cerebrospinal, or ascitic fluid). OM was defined as samples not meeting the PM criteria.

Patient features and classification in metastatic progression groups are further described in **Tables S1**, **S2** and **Figure S1**. All non-parametric Mann-Whitney tests (MWT) and Spearman correlations were conducted as two-tailed using GraphPad Prism software version 5.0 d (**Figure 1**; **Table S2**). Enrichment of phenotypes 2×2 contingency tables were conducted using two-tailed Fisher's Exact Test (FET). These metastatic progression sub-classifications were also reassessed in the independent validation samples of oligo- vs polymetastatic patients that we previously published [22] (**Table S3**, GSE25552).

### II. Tissue acquisition, RNA extraction and microRNA profiling

After Institutional Review Board approval, FFPE metastatic tissue samples were received in triplicate from the Department of Pathology at the University of Chicago. Total RNA was extracted from FFPE tissue samples using RecoverAll Total Nucleic Acid Isolation Kit (Applied Biosystems, Allston, MA, USA). Tissues of ≤80 µm were sectioned into sizes of 5–20 µm and underwent deparaffinization, protease digestion, nucleic acid isolation, and nuclease digestion/purification according to the manufacturer's protocol for RNA isolation. Sample concentrations were determined using the Qubit Quantification Platform (Invitrogen, Carlsbad, CA, USA) and normalized to 10 ng/µL. Ten µL of each triplicate were combined and 3 µL of this pooled sample were used to obtain a total of 30 ng of total RNA. Single stranded cDNA synthesis and pre-amplification were performed according to the manufacturer's protocols (Applied Biosystems, Allston, MA, USA). Real-time qPCR of 376 distinct microRNAs was performed using human Taqman MicroRNA Array A Card v2.0 (Applied Biosystems, Allston, MA) according to the manufacturer's protocol.

### III. Prioritization of deregulated microRNAs derived from oligo- and poly-metastatic lung metastasis patients and microRNA family analysis

RTqPCR SDS files for each patient sample were processed using RQManager v1.2.1 in order to obtain Ct (threshold cycle) values. The raw Ct values and array qualities were analyzed and normalized using HTqPCR package in Bioconductor. Sixty-three of the seventy-two human samples assayed by TaqMan microRNA Card A having sufficient clinical follow-up following last treatment and more than 230 detectable microRNAs (Ct<38) were included in the analysis. Four samples were excluded due to a lack of sufficient clinical follow-up, 3 other samples were excluded due to incomplete RTqPC reactions, and 2 other samples with less than 190 detectable microRNAs were also excluded (**Figure S2**). For the remaining 63 samples, the raw Ct values were normalized using the deltaCt method with the pooled mean of endogenous controls RNU-44 and RNU-48. RNU-44 and RNU-88 are two small non-coding RNA (ncRNAs) that are expressed both abundantly and stably that are widely used as endogenous controls for microRNA expression profiling normalization. The coefficient of variation (CV) of external and endogenous controls was ≤5% after normalization. The raw and normalized TaqMan array data of the lung metastasis clinical samples have been deposited in the NCBI GEO database with accession number GSE38698. Deregulated microRNA expression of LRP vs. HRP patient samples were “*prioritized*” using a two-tailed Student t-test at an unadjusted p-value<0.05 and organized according to their fold change (**Table 1**). Note that small sample sizes precluded achieving statistical significance after adjusting for multiple comparisons. Fold change values for microRNAs were calculated using the delta-delta CT method.

**Table 1:** Prioritized microRNAs between lung metastasis patients with a high rate or progression (HRP) and low rate of progression (LRP).

microRNA	Fold change	p-value
miR-654-5p	−8.06	0.0076
miR-655	−4.93	0.0066
miR-154	−4.87	0.0341
miR-329	−4.85	0.0231
miR-330-5p	−4.28	0.0037
miR-485-3p	−3.92	0.0177
miR-576-5p	−3.66	0.0091
miR-520a-3p	−3.58	0.0031
miR-127-5p	−3.47	0.0291
miR-380	−3.05	0.0123
miR-887	−2.97	0.037
miR-485-5p	−2.82	0.0194
miR-127-3p	−2.63	0.0172
miR-541	−2.56	0.0097
miR-453	−2.55	0.0082
let-7c	−2.51	0.0133
miR-369-3p	−2.47	0.022
miR-298	−2.45	0.0168
miR-299-3p	−2.36	0.0101
miR-582-5p	−2.3	0.0149
miR-153	−2.22	0.038
miR-544	−2.15	0.0253
miR-672	−2.12	0.0279
miR-296-3p	−2.12	0.0408
miR-448	−2.08	0.0455
miR-133a	−2.07	0.0384
miR-412	−2.03	0.0342
miR-328	−2	0.0427
miR-520g	−1.97	0.0471
miR-502-5p	−1.96	0.0534
miR-128	−1.95	0.0095
miR-199b-5p	−1.92	0.0538
let-7b	−1.87	0.0244
miR-135a	−1.86	0.0409
miR-199a-5p	−1.85	0.0518
miR-491-5p	−1.62	0.0359
miR-191	−1.41	0.0524
miR-506	4.15	0.0545
miR-205	4.55	0.0275
miR-98	11.14	0.0531

LRP patient n = 32; HRP patient n = 16. Fold change values for microRNAs were calculated using the delta-delta CT method, and p-values were calculated using a Student's t test (two-tailed p≤5%, **Methods**). A positive fold change represents elevated expression in patient samples with a high rate of progression as compared to a low rate of progression (LRP) progression (see **Tables S1** and **S2** for clinical information). Further details on the quality control for TaqMan microRNA expression is presented in **Figure S2**.

To analyze the enrichment of co-expressed microRNAs within microRNA families or a -3p/-5p microRNA expression pair within our prioritized list (**Table 1**), we conducted an empirical statistic as follows. All microRNAs were annotated for their hairpin sequence families by miRBase (miFam.dat, www.mirbase.org) [23]. In addition to microRNA families annotated in miRBase, we also included -3p/-5p complementary strand microRNA pairs in our family list in order to analyze their transcriptional co-regulation. Inclusion criteria for microRNA families required that sufficient microRNAs were available to evaluate the family in the Taqman array Card A (the minimum was set to three for microRNA families and to both members of the of -3p/-5p complementary microRNA strands grouping). In total, 15 distinct microRNA families and 50 pairs of -3p/-5p microRNAs met the inclusion criteria for the analysis. We thereafter conducted an empirical statistic to evaluate the significance of the total number of prioritized microRNA families and -3p/-5p expression pairs: (i) a microRNA family was prioritized by comparing microRNAs of the family with 2 fold-change of expression and unadjusted Student's t-test p≤0.05 to the remaining microRNAs in the family (families with odds ratio>3 of having at least 3 microRNAs congruently expressed with significant fold change in the same direction were counted as prioritized), and (ii) when both sense and antisense microRNA pairs (-3p and -5p suffixed pairs) were significantly and congruently co-expressed in the same direction with 2 fold-change of expression and unadjusted Student's t-test p≤0.05. We thereafter counted the sum of all prioritized microRNA families and of -3p/-5p pairs together in the observed set and compared them to an empirical set to determine the significance of this finding (reported in Table 2). We generated the empirical datasets by shuffling the sample labels (LRP or HRP) of all 63 samples into two groups of the same size for the LRP (n = 32) and HRP (n = 16) classifications and recalculating for each permutation the t-test of differentially expressed microRNAs at p<5% (10,000 permutation resamplings).

**Table 2:** Family and -3p/-5p co-expression analysis of microRNAs prioritized between metastatic samples of patients with high vs. low rates of progression.

Co-expressed microRNA family or -3p/-5p pairs	microRNA members
miR-154	miR-154
	miR-369-3p
	miR-655
miR-127	miR-127-3p
	miR-127-5p
miR-485	miR-485-3p
	miR-485-5p

Three microRNA families and -3p/-5p expression pairs were prioritized by enrichment in congruently co-expressed microRNA (empirical permutation resampling p = 0.0385, **Methods**).

### IV. Survival Analysis

**Figure 2:**
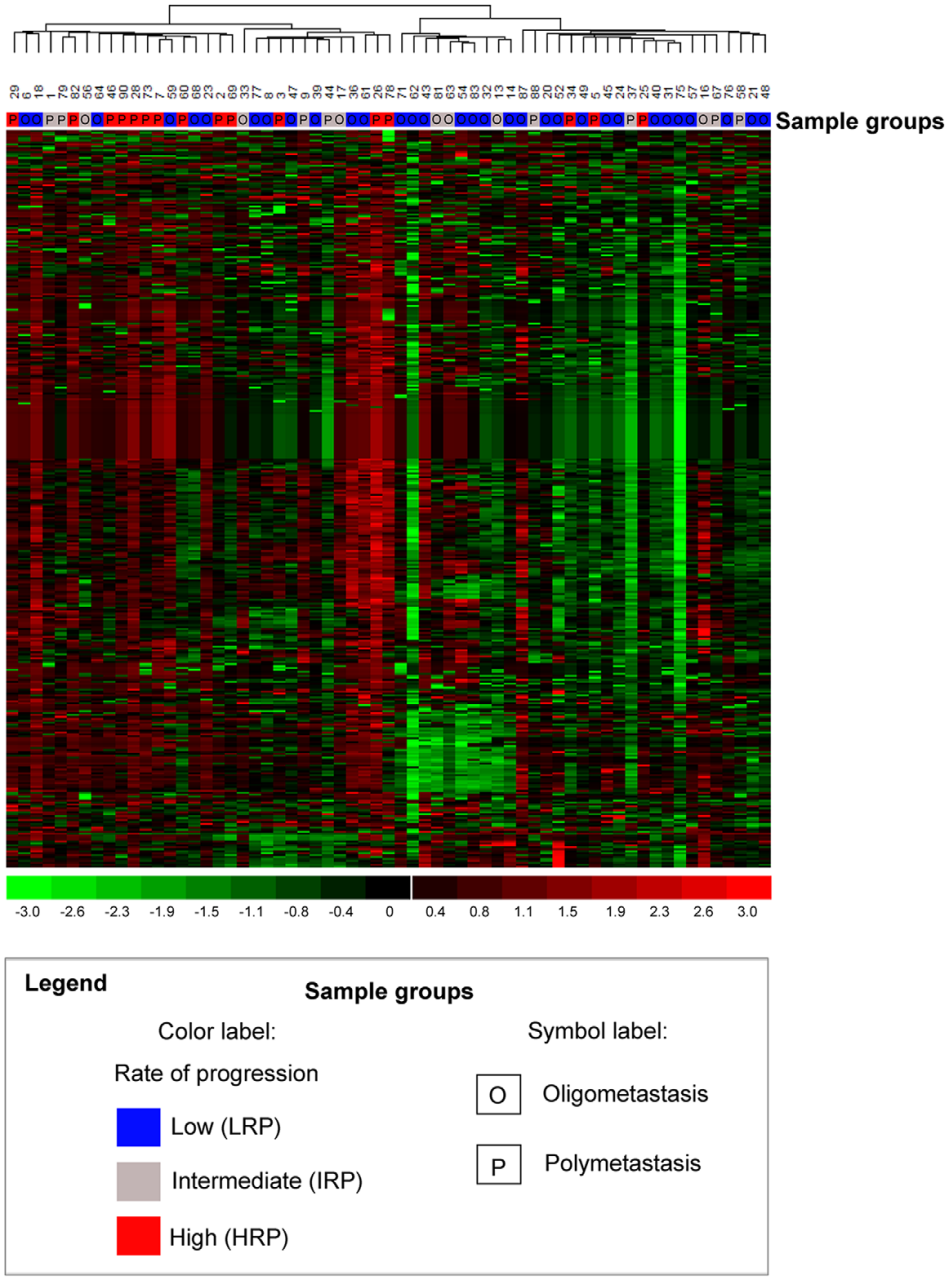
Unsupervised hierarchical clustering of microRNAs derived from lung metastasis samples distinguishes patients with high vs. low rates of metastatic progression. Expression of 384 microRNAs in metastatic lung tumor samples above, at, and below mean level are represented by red, black, and green TaqMan qPCR Ct values across all patients (n = 63) and were used to cluster oligo- and poly-metastatic patients. As shown above, 13 of 16 HRP patients (red on color-coded bar above the dendrogram) cluster together (left dendrogram branch) and 20 of 32 LRP patients (blue color-coded bar) cluster together from these HRP patients (right dendrogram branch), resulting in a divergence of these diametric sub-phenotypes (odds ratio = 7.22; Fisher's Exact Test, two-tailed p = 0.006). We also observed significant differences between patients classified as polymetastatic (P) vs. oligometastatic (O) (OR = 3.89, FET p = 0.019; **Methods**).

**Figure 3:**
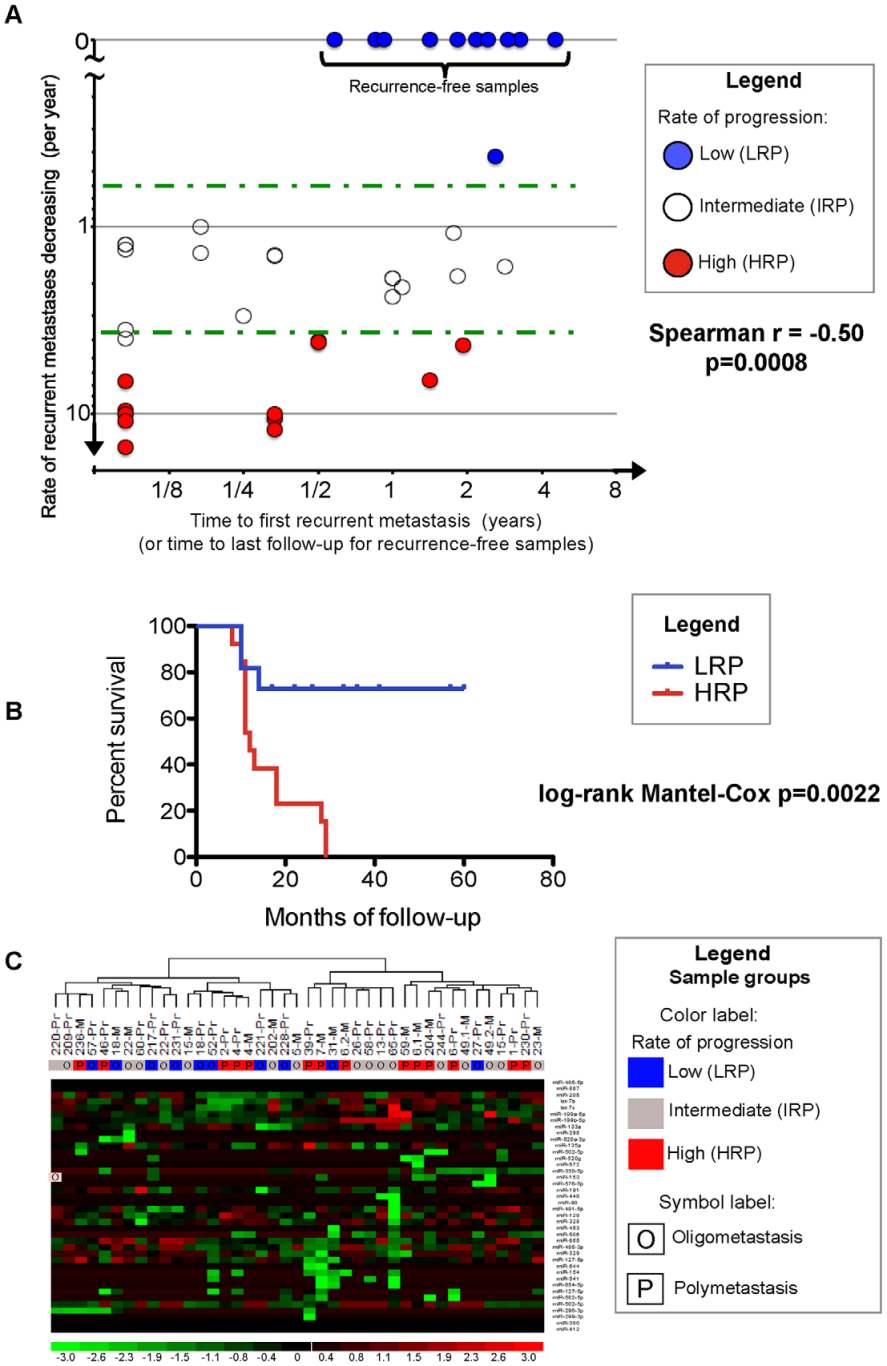
Rate of metastasis progression of patients in an independent validation dataset are associated to prioritized microRNAs discovered in patients with high vs. low rates of progression discovered in the lung metastasis dataset. **A**, patients of the independent validation dataset are stratified as described in Figure 1 according to a high rate of progression (HRP, red circles), low rate of progression (LRP, blue circles), and intermediate rate of progression (IRP, white circles). Green dotted lines represent rate thresholds. LRP patients exhibiting no recurrence after radiation therapy were also plotted as yielding 0 metastases/month on the y-axis. Of note, all HRP patients exhibit polymetastatic progression, all LRP patients exhibit oligometastatic progression, and IRP patients represent patients with oligometastatic progression that do not meet the LRP criteria (**Methods**). See **Table S3** for patient classification into metastatic progression groups in the independent validation dataset. **B**, Patients of the LRP (n = 10) and HRP (n = 14) subgroups from dataset GSE25552 were compared for their survival outcome using log-rank Mantel-Cox analysis. The median survival of LRP and HRP patients is 26 months and 12 months, respectively. A log-rank Mantel Cox p<0.0022 was obtained when comparing LRP vs. HRP survival outcome. **C**. microRNAs differentially expressed in HRP and LRP patients from the lung metastases samples stratify patients in independent dataset GSE25552. 8 of 10 LRP patients cluster together (left branch) and 9 of 14 HRP patients cluster separately from these LRP patients (right branch), resulting in a divergence of these metastatic phenotypes (odds ratio = 7.2; Fisher's Exact Test, two-tailed p = 0.047). Color-coded designations-see **Figure 1**.

Analysis of lung metastasis patient survival outcomes based on LRP vs. HRP classification (**Figures 2** **,** **3** **, S1** and **S4**) and all other survival analyses were calculated by the Log-rank Mantel-Cox test using GraphPad Prism software version 5.0 d. All non-parametric Mann-Whitney tests (MWT) and Spearman correlations were conducted as two-tailed using GraphPad Prism software version 5.0 d (**Figure 3**).

### V. Hierarchical clustering

Unsupervised hierarchical clustering with all 384 microRNAs of lung metastasis samples was conducted using dChip software with the default parameters “average” linkage and “1-Pearson” distance metric (**Figure 2**). Unsupervised hierarchical clustering using selected microRNA features (**Table 1**) within an independent validation study (**Figure 3**; GEO:GSE25552) was conducted using dChip with default parameters [22]. 5% to 95% confidence intervals of proportions were calculated using a Bayesian F-distribution [24]. Enrichment of phenotypes in the two main dendrogram branches of both hierarchical clustering analyses were respectively evaluated as 2×2 contingency tables for enrichment of HRP vs. LRP as well as OM vs. PM phenotypes using two-tailed Fisher's Exact Test (FET, **Figures 2** **,** **3**, and **4**).

**Figure 4:**
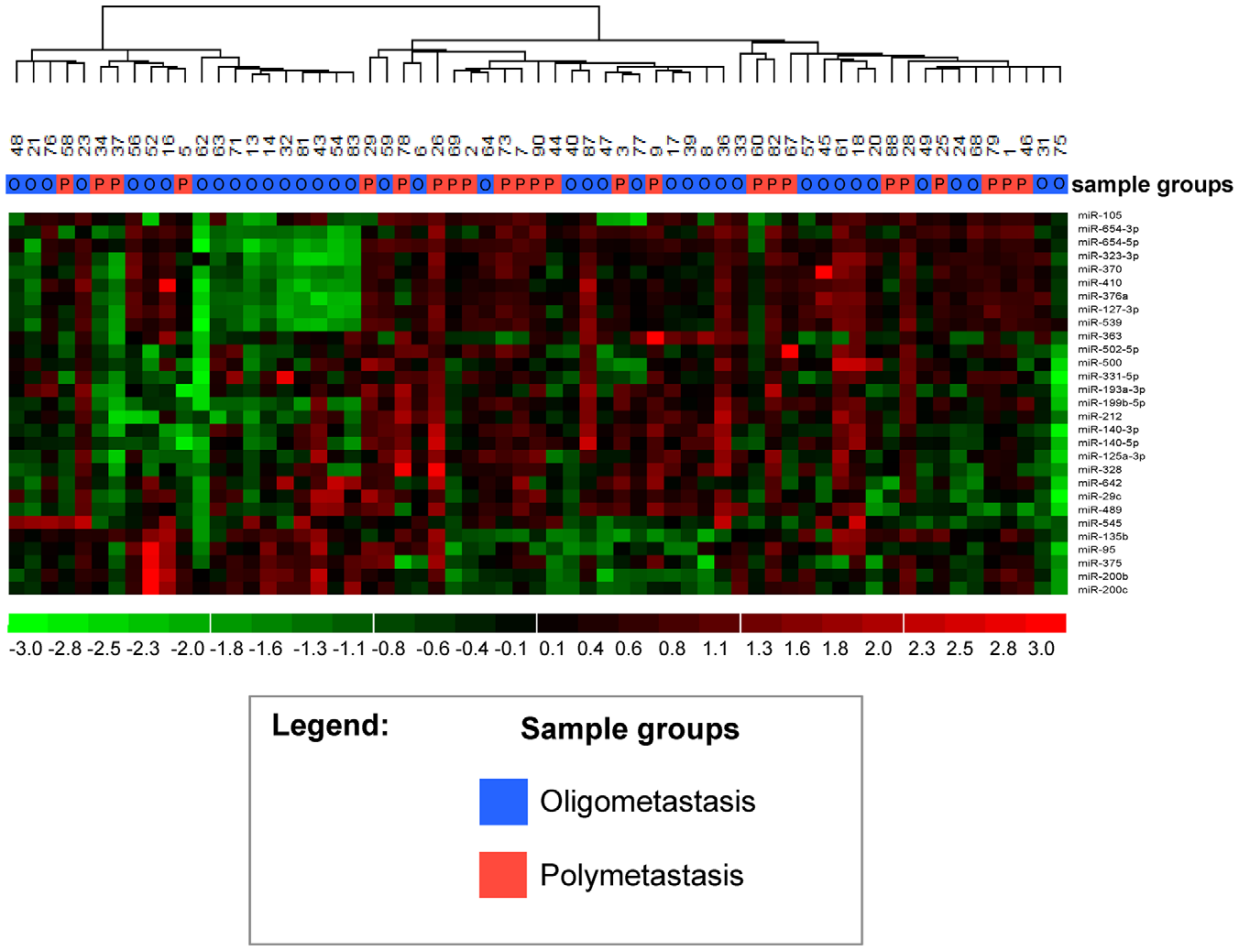
Lung oligometastatic and polymetastatic progression samples are differentiated through hierarchical clustering with prioritized microRNA features derived from the independent validation dataset GSE25552. 29 microRNAs differentially expressed between polymetastatic vs. oligometastatic progression (GSE25552) were used to stratify lung metastatic samples, described in the current report (n = 63) [22]. As shown above, 17 of 39 oligometastatic progression patients cluster together (left branch) and 20 of 24 polymetastatic progression patients cluster separately (right branch; odds ratio = 3.86; Fisher's Exact Test, two-tailed p = 0.032; **Methods**). See **Methods** for definitions of polymetastatic and oligometastatic progression.

### VI. Preparation of independent validation study samples

Independent microRNA expression data used in our meta-analysis (**Figure 3**) were previously published and are deposited in GEO:GSE25552 [22]. We used the Bioconductor HTqPCR package to assess the quality of the arrays and normalize the raw Ct (threshold cycle) data of all patient samples (n = 45). Three samples with less than 190 detectable microRNAs were excluded (**Figure S3**). A principal component analysis (PCA) plot of the 1^st^ and 2^nd^ components revealed no batch effect between primary and metastatic samples using the plotCtPCA function in the HTqPCR package. The probes were flagged based on their Ct value: below 10 were flagged as “Unreliable”; above 37 were flagged as “Undetermined”. The remaining Ct values were flagged as “OK” and normalized using quantile normalization implemented in the normalizeCtData function of Bioconductor. Patient sample “22c” was removed due to a lack of clinical phenotype verification [22].

## Results

### I. Stratification of patients according to the rate of lung metastasis(es) progression

Sixty-three patients met the criteria for analysis, including pathological confirmation and clinical follow-up. Stratification of the three metastatic progression phenotypes (HRP, LRP, IRP; **Methods**) is shown in **Figure 1A**, using the rate of progression and the time to first metastatic recurrence after resection. A binary oligometastases (OM) and polymetastases (PM) classification as previously described [22] was applied for further comparison with the independent dataset. Of note, all HRP patients were classified as PM, all LRP patients were classified as OM, and IRP patients include both types (**Methods**). We found a strong negative correlation between the rate of recurrent metastases with the time to first recurrence following metastasectomy (n = 63, Spearman r = −0.90, p<0.0001). A difference in survival was observed between the LRP and HRP subset of patients (n = 48, log-rank Mantel-Cox test p<0.0001), as presented in **Figure 1B**. LRP patients developed fewer recurrent metastases following surgery compared to HRP patients, (p<0.0001, two-tailed MWT; **Table S2**, Panel A). Patients were more likely to remain alive throughout follow-up when classified in the LRP group (two-tailed p = 0.0001, **Table S2**, Panel A). Similar results were found when comparing OM vs. PM patient groups (**Table S2**, Panel B). We also found a correlation between the rate of recurrent metastasis(es) and the number of metastasis(es) present one year post-surgery in LRP and HRP patients (n = 48, nonparametric Spearman r = 0.88, two-tailed p<0.0001).

Clinical features for metastatic progression groups are shown in **Table S2**. There was no difference between LRP and HRP patients in (i) age at time of surgery (ii) the number of metastases at the time of surgery or (iii) the proportion of histological types (e.g. sarcomas, adenocarcinomas) (**Table S2**, Panel A; MWT pvalues not significant). We observed improved survival in adenocarcinomas compared to non-adenocarcinomas; however, the HRP and LRP phenotype remained significant for all histological types (**Figure S1**). The initial number of metastases did not correlate with survival (Spearman r = −0.12, two-tailed p = 0.35 *ns*; log-rank Mantel-Cox test p = 0.54 *ns*).

### II. microRNAs associated with rate of progression of lung metastases

As shown in Figure **2**, the first dendrogram branch of an unsupervised hierarchical clustering of lung metastasis patient samples using 384 microRNAs assayed distinguished LRP from HRP patients (odds ratio = 7.22; two-tailed FET p = 0.006), recalled 13 of 16 HRP patients (81%; left dendogram branch; 5% to 95% confidence intervals: 56% to 93%) and discriminated the LRP phenotype more precisely than the HRP (87%; right dendogram branch comprising 20 LRP & 3 HRP, 5% to 95% confidence intervals: 68% to 95%). We also found that LRP and HRP clusters were enriched by OM and PM patients respectively, using our previous classification (odds ratio = 3.89, FET p = 0.019, **Figure 2**). In order to identify microRNA expression patterns associated with the most distinct phenotypes of metastatic progression, we identified 40 microRNAs differentially expressed (p≤0.05, Student-t test, n = 48 samples) between the LRP and HRP subgroups using delta-Ct normalization against endogenous microRNA controls RNU-44 and RNU-48 (**Table 1**). A large number of the prioritized microRNAs were down-regulated in HRP relative to LRP patients and have been implicated in tumor suppression functions (e.g. let-7 family members, **Table S4**). Further, in order to validate that these prioritized, differentially expressed microRNAs are biologically congruent; we analyzed their common features. MicroRNA families as well as -3p and -5p (-3p/-5p) strands expression pairs [25] were prioritized in the lung dataset by enrichment of congruently co-expressed microRNAs. Three groups of microRNAs were found consistently deregulated (**Table 2**, empirical permutation resampling p = 0.0385). One microRNA family (miR-154) and two -3p/-5p pairs of microRNA strands (miR-127 and miR-485) were significantly enriched compared to expected ratios (permutation resampling p = 0.0385; **Table 2** **; Methods**).

### III. Validation of prioritized microRNAs associated with the rate of progression in an independent dataset

In order to validate the prioritized microRNAs derived from the lung metastasis dataset, we tested capability of LRP vs. HRP classification in a previously reported independent dataset [22] (GSE25552, **Methods**). We classified patients of GSE25552 dataset according to the proposed LRP and HRP threshold criteria (**Table S3**). Consistent with the present study, there was no significant difference in the number of initial metastases between LRP and HRP patients and no correlation between the initial number of metastases and survival. In addition, we report a negative correlation between the rate of recurrent metastases and the time to first recurrence (n = 41, Spearman r = −0.50, p = 0.0008, **Figure 3A**). Survival was significantly different when comparing HRP vs. LRP patients (n = 24, log-rank Mantel-Cox p = 0.0022, **Figure 3B**). Selecting the 40 microRNAs prioritized between LRP vs. HRP lung metastases patients (listed in **Table 1**), we performed an unsupervised hierarchical clustering analysis of their expression in the independent validation dataset. This approach resulted in a divergence of LRP and HRP patients (n = 41, odds ratio = 7.2; two-tailed FET p = 0.047, **Figure 3C** **; Methods**, **Table S3**). Clustering did not show separation between primary vs. metastatic tumors (n = 41, two-tailed FET p = 0.53 *ns*), suggesting that the selected microRNAs characterize both primary and metastatic tumors. Since 21 patients had no metastases within the LRP group while 11 had metastases, we hypothesized that patients with no metastases may have had defective abilities to colonize the lung. We compared the expression of microRNAs of these LRP subgroups to test this hypothesis and did not identify differentially expressed ones (data not shown).

### IV. Co-expression of microRNAs families and microRNAs overlapping between two independent datasets

We conducted two types of comparisons between the microRNAs reported here (**Table 1**) and those previously reported by us. First, differentially expressed microRNAs between LRP and HRP samples of the lung metastases (37 down-regulated microRNAs in HRP samples, **Table 1**) were compared to those we previously reported as oligometastatic and polymetastatic classification [22]. Three overlapping microRNAs with the same fold change direction were identified: miR-199b-5p, miR-328, and miR-502-5p. Second, we further annotated the samples of the GSE25552 dataset based on the rate classification described in the current report (**Figure 3**) and recalculated differentially expressed microRNAs. 21 MicroRNAs down regulated in HRP vs. LRP phenotypes (GSE25552) were compared with those described in **Table 1** of the current report. Using this comparison, we identified miR-328, miR-502-5p, and miR-491-5p as overlapping in both. Note that miR-328 and miR-502-5p were identified in both comparisons. Finally, we also confirmed that the microRNA features previously prioritized between OM vs. PM metastatic samples in the independent validation dataset can classify patients exhibiting OM vs. PM progression in the lung metastases samples in this study (OR = 3.83, p = 0.032, **Figure 4**).

## Discussion

In patients resected with limited pulmonary metastases, we identified differential microRNA expression patterns between patients with low and high rates of metastatic progression. We validated the ability of the progression phenotypes to predict survival (**Figure 1B** and **3B**), and then demonstrated that the prioritized microRNAs were able to predict phenotypes in two datasets [22]. These results support our hypothesis of oligometastasis as a clinical entity with biological mechanisms that may differ from polymetastatic disease.

Preclinical and clinical data support differential metastatic potential amongst tumors cells [26]–[28]. Our current analysis, based on the rate of progression of metastasis emphasizes growth properties of different metastatic clones [29]–[32]. However, differences in the ability to colonize the lung microenvironment following primary tumor resection may also account for observed differences between oligo- and poly- metastatic phenotypes [33].

During the metastatic cascade, cancer cells acquire properties to colonize distant sites [34], [35]. The acquisition of mutations that enable metastasis has been shown to be hierarchical, with a long preclinical phase [36]. Loss or deficiency in any portion of the cascade could reduce metastatic potential. Host and tumor factors affecting tumor dormancy, growth rate, and colonization are also important in the evolution of metastasis [37]. Considered together with our data, these reports provide a rationale for understanding the biological and clinical basis of oligometastases.

The microRNAs prioritized between patients with a high vs. low rate of recurrent metastasis in the independent dataset (**Figure 3C**) suggest distinct forms of metastatic progression while accurately predicting survival (**Figure 3B**). Our proposed classification of discrete progression groups is a conservative method of analysis. More samples per class are required to reach significance as compared to a single continuous phenotype analysis. The prioritized microRNA features (**Table 1**) should be further tested and refined in larger cohorts of patients. A larger cohort would permit analysis of low vs no metastasis patients (**Figure 1**), which did not reveal differences in microRNA expression (data not shown).

MicroRNAs have an important role in cancer. They are known to control cell proliferation and apoptosis [38]. Also, malignant tumors and cell lines have deregulated microRNA compared with normal tissue [39]. In this study, we compared lists of differentially expressed microRNAs between our two independent datasets, using both the rate of progression phenotype described here and the binary OM/PM phenotypes. MiR-328 and miR-502-5p overlapped in both comparisons and under-expressed in HRP and PM patient samples; this aligns with the finding that widespread reduction in microRNA expression promotes tumorigenesis [40]. Although miR-502-5p has not been well characterized with respect to cancer, miR-328 has been shown to function as an RNA decoy to interfere with the function of cell regulatory proteins in chronic myelogenous leukemia (CML). Loss of miR-328 was shown to occur in the blast crisis of chronic CML, and restoration of its expression diminished the survival of leukemic blasts [41]. Furthermore, the majority of deregulated microRNAs detected in this study is down-regulated in HRP patients and are associated with tumor-suppression functions (**Table 1**, **Table S4**). Although their role is not yet clear, these microRNAs provide a starting point for further investigation of molecular pathways discriminating oligo- and poly- metastatic phenotypes.

Only two microRNAs overlapped between two independent datasets. This small overlap is consistent with reports that gene signatures developed in different cohorts may share minimal overlap of features, despite the fact they are predictive of the same clinical outcomes [42], [43], [44]. It has been postulated that different molecular features among signatures (e.g. genes, microRNAs) may represent shared pathways or mechanisms that can convey similar outcomes. We and others have shown that pathway-based approaches to analyzing genetic signatures predictive of clinical outcome are more reliable than traditional gene-based methods [45], [46], [47]. We analyzed microRNA families collected in miRBase to assess the prevalence of microRNA families, as defined by their shared transcriptional and host gene regulation (**Methods**), in our list of differentially expressed microRNAs in lung metastasis samples [23]. We focused our family analysis of prioritized microRNAs between HRP vs. LRP patients of the lung metastasis samples in this study and present the results in **Table 2**. Notably, three members of the mir-154 family were identified as congruently down-regulated in HRP samples as compared to LRP samples in the lung metastasis dataset. miR-154 has been reported to suppress tumor cell growth in the G(1)/S [48].

This report provides additional evidence that a substantial subset of patients with limited metastases at first metastatic presentation may controlled with local therapies of curative intent [49]. Our data also support the feasibility of improving the accuracy of microRNA classifiers predictive of therapeutic response to local therapies among these patients. By targeting curative intent local treatments of metastases to the subset of patients with predicted oligometastasis progression, microRNA classifiers can provide a rational criterion for offering these therapies. One limitation of our data is the relatively small numbers of patients, thus larger cohorts of patients will be required to further increase the accuracy of the microRNA classifier in preparation of clinical trials. However, the two datasets described herein are the only known clinically and molecularly classified sets of patients with oligometastases. Also, we emphasize the importance of rate of recurrence, however some patients in the LPR group had no recurrences at the time of analysis and therefore the microRNAs identified may reflect lung colonization. These two concepts may be intertwined in our analysis and further laboratory and clinical analysis is necessary to identify the contribution of these variables. In summary, the presented data support the hypothesis that different biological mechanisms underlie lung oligo- and poly- metastatic progression. These processes likely reflect lung colonization and growth rate of established metastases.

## Supporting Information

Figure S1 **Adenocarcinoma histology survival analysis of HRP and LRP patients.** We observed a difference in survival outcome between patients of the lung metastasis dataset (n = 63) with adenocarcinoma primary histology (n = 22) vs. other histological types (n = 41) using a log-rank Mantel-cox test (p<0.009). Therefore, we investigated whether this confounding effect in survival outcome could be observed between HRP vs. LRP patients. We found that HRP vs. LRP classification remained significant in terms of survival outcome between patients with adenocarcinoma and non-adenocarcinoma primary histologies. In **Panel A**, lung metastasis patients with adenocarcinoma primary histologies of the LRP (n = 16) and HRP (n = 3) subgroups were compared for their survival outcome using log-rank Mantel-Cox analysis. A log-rank Mantel Cox p<0.0001 was obtained when comparing LRP vs. HRP survival outcome. In **Panel B**, lung metastasis patients with non-adenocarcinoma primary histologies of the LRP (n = 16) and HRP (n = 13) subgroups were compared for their survival outcome using log-rank Mantel-Cox analysis. A log-rank Mantel Cox p<0.0001 was obtained when comparing LRP vs. HRP survival outcome.
(PDF)

Figure S2 **Quality control measurement of microRNAs in each lung metastasis patient sample.** To control for microRNA quality, the number of total detectable microRNAs per sample (n = 65 samples) was plotted using the Bioconductor package HTqPCR. For samples to be included in this study, we required that at least 230 detectable microRNAs could be detected. Patient IDs 65c and 89c were excluded due to their excessive number of undetermined microRNAs.
(PDF)

Figure S3 **Quality control measurement of microRNAs in the primary and metastatic patient samples of the independent validation dataset.** To control for microRNA quality, the number of total detectable microRNAs per sample (n = 45 samples) was plotted using the Bioconductor package HTqPCR. For samples to be included in this study, we required that at least 180 detectable microRNAs could be detected. Patient IDs 49b, 15c and 5a were excluded due to their excessive number of undetermined microRNAs.
(PDF)

Table S1 Description of clinical features of lung metastasis patients. For each patient in this study, the following clinical information is provided and organized by metastatic rate phenotype: Patient ID; Gender; Primary type; Primary histology; Number of metastases at time of surgery; Progression of metastases within a body cavity status (Yes/No); Total number of recurrent metastases (following surgery); Time from surgery to first metastatic recurrence (months); Rate of recurrent metastases (per month) between lung surgery and time to last follow-up; Alive status (Yes/No); Survival (months); Metastatic rate phenotype (HRP, LRP, IRP); Oligo- vs. poly- metastatic progression (OM, PM).
(PDF)

Tables S2 Characteristics of patients with LRP and HRP progression (S2A) and oligometastatic (OM) and polymetastatic (PM) progression (S2B) in lung metastasis samples. Legend: two-tailed Fisher's Exact Test (FET); two-tailed non-parametric Mann Whitney Test (MWT); log-rank Mantel-Cox test (Log-rank); * = statistically significant.
(PDF)

Table S3 Classification of primary and metastatic patient samples of the independent validation dataset into stratified metastatic phenotypes. For each patient in the independent validation study [GEO: GSE25552], the following clinical information is provided and organized by metastatic rate phenotype: Patient ID; Primary vs. metastasis tumor type; Time to first metastatic recurrence following radiotherapy (months); Rate of recurrent metastasis(es) per month following radiotherapy throughout follow-up, Alive status (Yes/No); Survival (months); Metastatic rate phenotype (HRP, LRP, IRP); Oligo- vs. poly-metastatic progression (OM, PM). Note for this study that all HRP patients must also be classified as PM and all LRP patients must be classified as OM. The * in the Survival (months) column represents updated follow-up since the original publication [22].
(PDF)

Table S4 Evidence of tumor suppression and tumor promotion functions of prioritized microRNAs between LRP and HRP patients of the lung metastasis dataset. The tumor suppressing and tumor promoting functions of each of the 40 prioritized microRNAs between LRP and HRP patients of the lung metastasis dataset (Table 1) were investigated. Inclusion criteria used for citations required that each study provide experimental evidence for the role of the microRNA in a cancer context (i.e. cancer cell culture model, animal model of cancer, or human cancer samples) and that functional assays were performed. For instance, differential expression of a microRNA between cancer and control states were not considered as experimental evidence if they were not accompanied with experiments examining the functional role in either suppressing or promoting the cancer phenotype in regards to the expression results. Experiments conducted in human samples were considered if expression results were correlated to meaningful clinical variables (e.g. survival outcome, metastatic progression).
(PDF)
