# Correction: Oligo- and Polymetastatic Progression in Lung Metastasis(es) Patients Is Associated with Specific MicroRNAs

**DOI:** 10.1371/annotation/2489ae5e-3650-4897-8df6-3e974ca585c4

**Published:** 2013-06-07

**Authors:** Yves A. Lussier, Nikolai N. Khodarev, Kelly Regan, Kimberly Corbin, Haiquan Li, Sabha Ganai, Sajid A. Khan, Jennifer L. Gnerlich, Thomas E. Darga, Hanli Fan, Oleksiy Karpenko, Philip B. Paty, Mitchell C. Posner, Steven J. Chmura, Samuel Hellman, Mark K. Ferguson, Ralph R. Weichselbaum

The eighth author's name was spelled incorrectly. The correct name is: Jennifer L. Gnerlich. 

