# Supplementary material for: Oligo- and Polymetastatic Progression in Lung Metastasis(es) Patients Is Associated with Specific MicroRNAs
Source: PLoS One. 2012 Dec 10;7(12):e50141. doi: 10.1371/journal.pone.0050141 (PMC3518475; doi:10.1371/journal.pone.0050141)
Supplement: Figure S1 — Adenocarcinoma histology survival analysis of HRP and LRP patients. We observed a difference in survival outcome between patients of the lung metastasis dataset (n = 63) with adenocarcinoma primary histology (n = 22) vs. other histological types (n = 41) using a log-rank Mantel-cox test (p<0.009). Therefore, we investigated whether this confounding effect in survival outcome could be observed between HRP vs. LRP patients. We found that HRP vs. LRP classification remained significant in terms of survival outcome between patients with adenocarcinoma and non-adenocarcinoma primary histologies. In Panel A, lung metastasis patients with adenocarcinoma primary histologies of the LRP (n = 16) and HRP (n = 3) subgroups were compared for their survival outcome using log-rank Mantel-Cox analysis. A log-rank Mantel Cox p<0.0001 was obtained when comparing LRP vs. HRP survival outcome. In Panel B, lung metastasis patients with non-adenocarcinoma primary histologies of the LRP (n = 16) and HRP (n = 13) subgroups were compared for their survival outcome using log-rank Mantel-Cox analysis. A log-rank Mantel Cox p<0.0001 was obtained when comparing LRP vs. HRP survival outcome. (PDF) [file pone.0050141.s001.pdf]

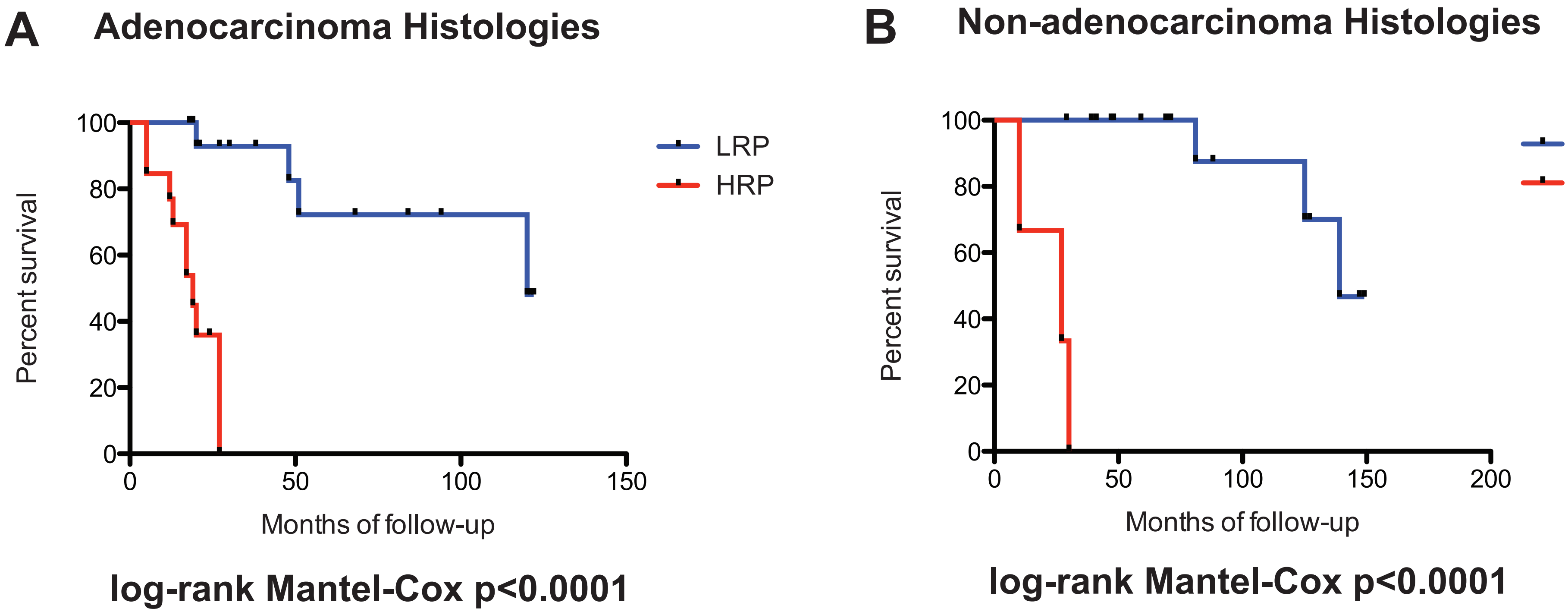

Supplementary Figure S1. Adenocarcinoma histology survival analysis of HRP and LRP patients. We observed a significant difference in survival outcome between all patients of the lung metastasis dataset (n=63) with adenocarcinoma primary histology (n=22) vs. other histology types (n=41) using a log-rank Mantel-cox test (p<0.009). Therefore, we investigated whether this confounding effect in survival outcome could be observed between HRP vs. LRP patients. We found that indeed HRP vs. LRP classification remained significant in terms of survival outcome between patients within the adenocarcinoma histology as wel as within the non-adenocarcinoma primary histology. In **Panel A**, lung metastasis patients with adenocarcinoma primary histologies of the LRP (n=16) and HRP (n=3) subgroups were compared for their survival outcome using log-rank Mantel-Cox analysis. A log-rank Mantel Cox p<0.0001 was obtained when comparing LRP vs. HRP survival outcome. In **Panel B**, lung metastasis patients with non-adenocarcinoma primary histologies of the LRP (n=16) and HRP (n=13) subgroups were compared for their survival outcome using log-rank Mantel-Cox analysis. A log-rank Mantel Cox p<0.0001 was obtained when comparing LRP vs. HRP survival outcome.
