# Supplementary material for: Oligo- and Polymetastatic Progression in Lung Metastasis(es) Patients Is Associated with Specific MicroRNAs
Source: PLoS One. 2012 Dec 10;7(12):e50141. doi: 10.1371/journal.pone.0050141 (PMC3518475; doi:10.1371/journal.pone.0050141)
Supplement: Figure S2 — Quality control measurement of microRNAs in each lung metastasis patient sample. To control for microRNA quality, the number of total detectable microRNAs per sample (n = 65 samples) was plotted using the Bioconductor package HTqPCR. For samples to be included in this study, we required that at least 230 detectable microRNAs could be detected. Patient IDs 65c and 89c were excluded due to their excessive number of undetermined microRNAs. (PDF) [file pone.0050141.s002.pdf]

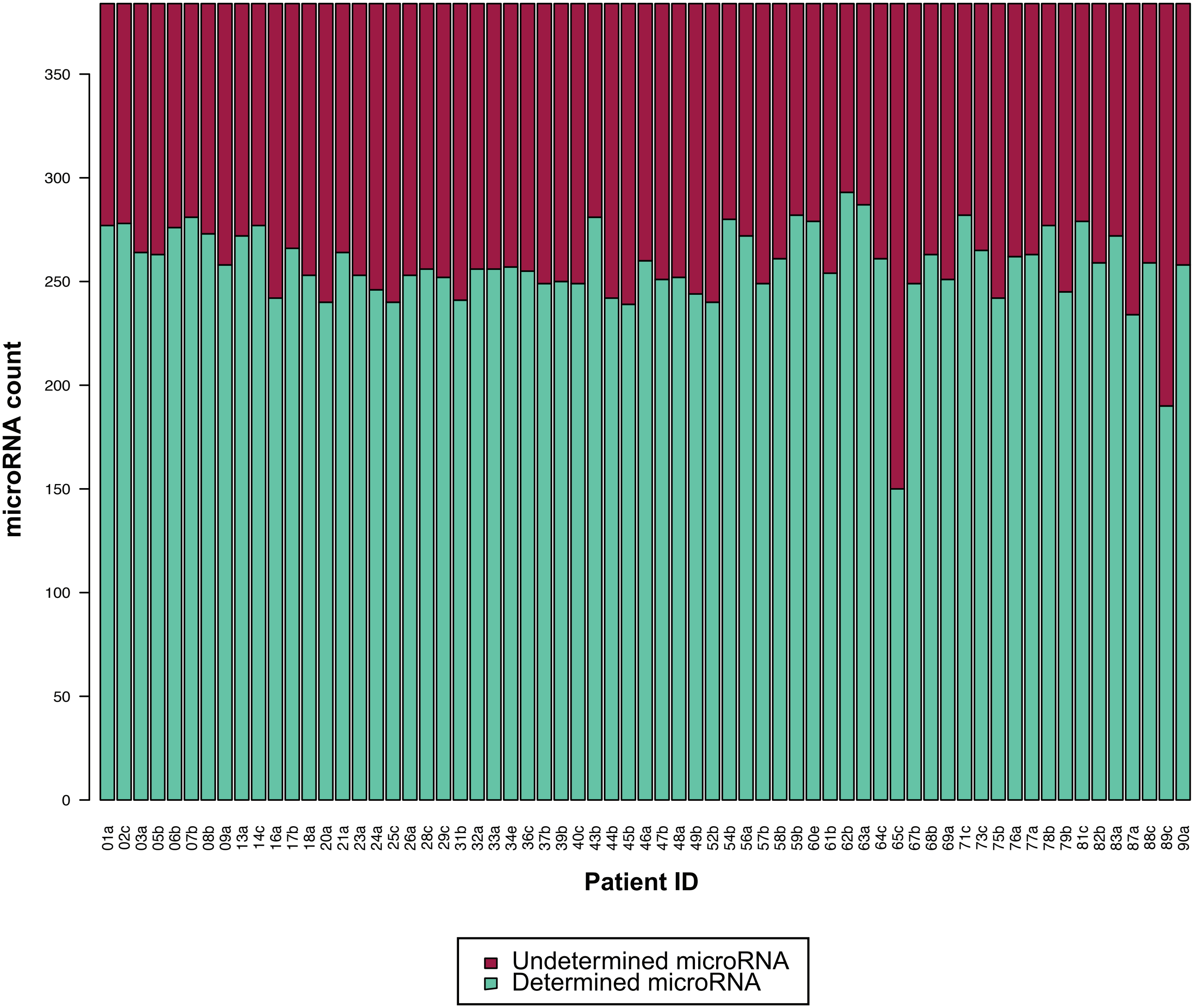

Supplementary Figure S2. Quality control measurement of microRNAs in each lung metastasis patient sample. To control for microRNA quality, the number of total detectable microRNAs per sample (n=65 samples) was plotted using the Bioconductor package HTqPCR. For samples to be included in this study, we required that at least 230 detectable microRNAs could be detected. Patient IDs 65c and 89c were excluded due to their excessive number of undetermined microRNAs.
