# Supplementary material for: Oligo- and Polymetastatic Progression in Lung Metastasis(es) Patients Is Associated with Specific MicroRNAs
Source: PLoS One. 2012 Dec 10;7(12):e50141. doi: 10.1371/journal.pone.0050141 (PMC3518475; doi:10.1371/journal.pone.0050141)
Supplement: Figure S3 — Quality control measurement of microRNAs in the primary and metastatic patient samples of the independent validation dataset. To control for microRNA quality, the number of total detectable microRNAs per sample (n = 45 samples) was plotted using the Bioconductor package HTqPCR. For samples to be included in this study, we required that at least 180 detectable microRNAs could be detected. Patient IDs 49b, 15c and 5a were excluded due to their excessive number of undetermined microRNAs. (PDF) [file pone.0050141.s003.pdf]

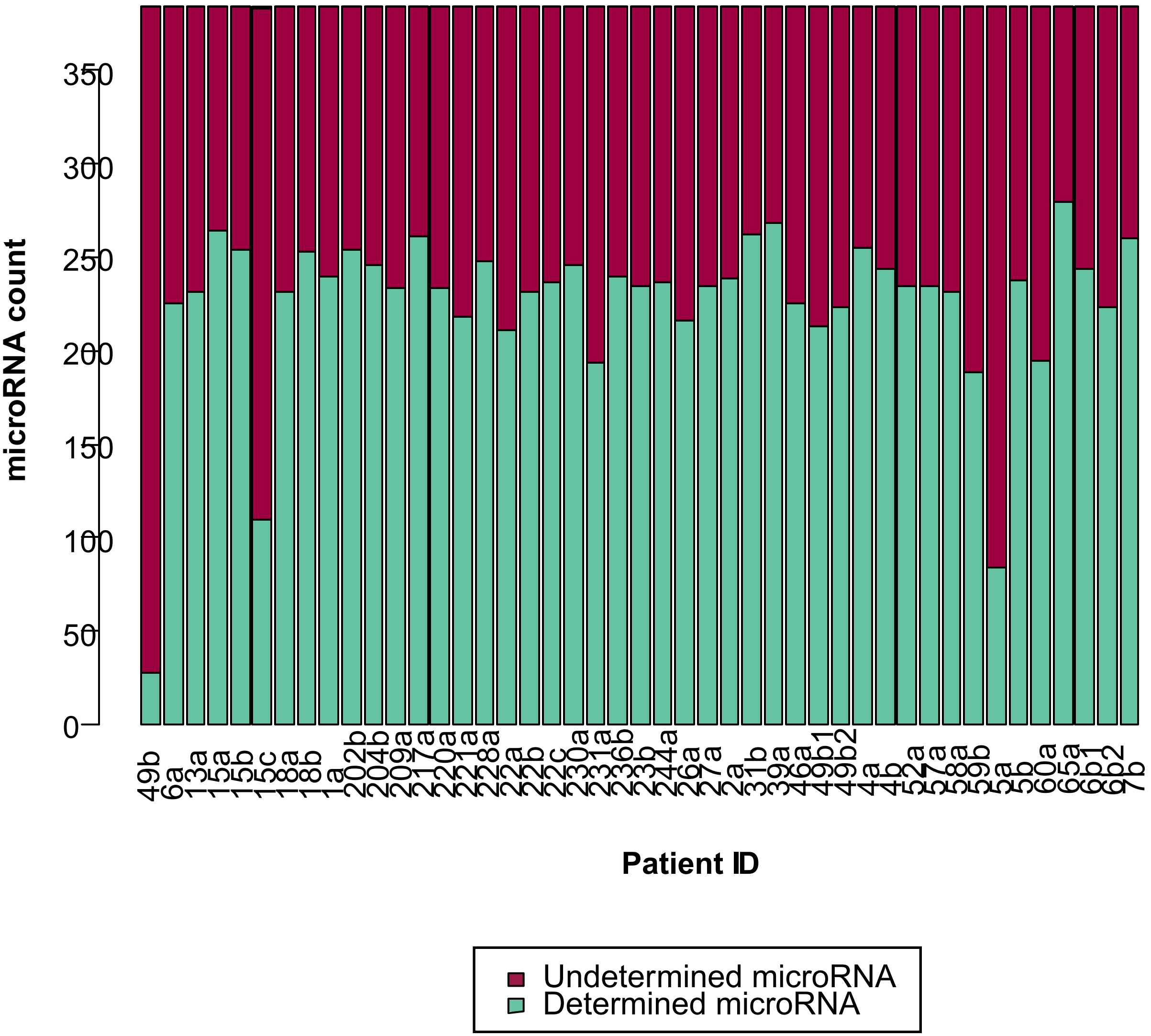

Supplementary Figure S3. Quality control measurement of microRNAs in the primary and metastatic patient samples of the independent validation dataset. To control for microRNA quality, the number of total detectable microRNAs per sample (n=45 samples) was plotted using the Bioconductor package HTqPCR. For samples to be included in this study, we required that at least 180 detectable microRNAs could be detected. Patient IDs 49b, 15c and 5a were excluded due to their excessive number of undetermined microRNAs.
