# Supplementary material for: Oligo- and Polymetastatic Progression in Lung Metastasis(es) Patients Is Associated with Specific MicroRNAs
Source: PLoS One. 2012 Dec 10;7(12):e50141. doi: 10.1371/journal.pone.0050141 (PMC3518475; doi:10.1371/journal.pone.0050141)
Supplement: Table S1 — Description of clinical features of lung metastasis patients. For each patient in this study, the following clinical information is provided and organized by metastatic rate phenotype: Patient ID; Gender; Primary type; Primary histology; Number of metastases at time of surgery; Progression of metastases within a body cavity status (Yes/No); Total number of recurrent metastases (following surgery); Time from surgery to first metastatic recurrence (months); Rate of recurrent metastases (per month) between lung surgery and time to last follow-up; Alive status (Yes/No); Survival (months); Metastatic rate phenotype (HRP, LRP, IRP); Oligo- vs. poly- metastatic progression (OM, PM). (PDF) [file pone.0050141.s004.pdf]

Supplementary Table S1. Description of clinical features of lung metastasis patients. For each patient in this study, the following clinical information is provided and organized by metastatic rate phenotype: Patient ID; Gender; Age (years) at lung surgery; Primary type; Primary histology; Number metastases at time of surgery; Progression of metastases within a body cavity status (Yes/No); Total number of recurrent metastases (following surgery); Time from surgery to first metastatic recurrence (months); Rate of recurrent metastases (per month) between lung surgery and time to last follow-up; Alive status (Yes/No); Survival (months); Metastatic rate phenotype: HRP, LRP, IRP; Oligo- vs. poly- metastatic progression: OM, PM.

Patient ID	Gender	Age (years) at lung surgery	Primary type	Primary histology	Number metastases at time of surgery	Progression of metastases within a body cavity	Total number of recurrent metastases	Time from surgery to first metastatic reccurence (months)	Rate of recurrent metastasis (per month)	Alive	Survival (months)	Metastatic rate phenotype	Oligo- vs. poly- metastatic phenotype
6	F	53	Breast	Adenocarcinoma	2	No	0	N/A	0.00	Yes	48	LRP	OM
8	M	57	Colon	Adenocarcinoma	2	No	2	53	0.03	Yes	59	LRP	OM
14	F	56	Extremity	Soft tissue sarcoma	2	No	1	67	0.01	Yes	68	LRP	OM
18	F	60	Breast	Adenocarcinoma	1	No	1	74	0.01	Yes	88	LRP	OM
20	F	62	Breast	Adenocarcinoma	1	No	0	N/A	0.00	No	139	LRP	OM
21	F	64	Uterus	Uterine sarcoma	1	No	0	N/A	0.00	Yes	51	LRP	OM
23	F	79	Colon	Adenocarcinoma	1	No	0	N/A	0.00	Yes	39	LRP	OM
24	F	46	Breast	Adenocarcinoma	2	No	0	N/A	0.00	Yes	149	LRP	OM
31	M	57	Colon	Adenocarcinoma	1	No	0	N/A	0.00	Yes	71	LRP	OM
32	F	74	Extremity	Soft tissue sarcoma	1	No	0	N/A	0.00	Yes	27	LRP	OM
36	M	63	Bladder	Adenocarcinoma	2	No	0	N/A	0.00	Yes	47	LRP	OM
39	F	45	Colon	Adenocarcinoma	2	No	0	N/A	0.00	Yes	127	LRP	OM
40	M	47	Colon	Adenocarcinoma	1	No	0	N/A	0.00	Yes	147	LRP	OM
43	M	36	Liver	Hepatocellular carcinoma	1	No	0	N/A	0.00	Yes	19	LRP	OM
45	F	67	Thyroid	Adenocarcinoma	2	No	3	60	0.02	No	125	LRP	OM
47	M	32	Colon	Adenocarcinoma	1	No	0	N/A	0.00	No	81	LRP	OM
48	F	58	Extremity	Soft tissue sarcoma	1	No	0	N/A	0.00	Yes	84	LRP	OM
49	F	69	Renal	Renal cell carcinoma	1	No	0	N/A	0.00	Yes	122	LRP	OM
52	F	55	Uterus	Soft tissue sarcoma	1	No	2	6	0.04	No	48	LRP	OM
54	M	26	Mesentary	Soft tissue sarcoma	2	No	2	14	0.04	No	51	LRP	OM
57	F	58	Sacrum	Soft tissue sarcoma	1	No	0	N/A	0.00	Yes	94	LRP	OM
59	F	78	Cervix	Adenoid cystic carcinoma	1	No	0	N/A	0.00	Yes	30	LRP	OM
61	M	82	Prostate	Adenocarcinoma	2	No	0	N/A	0.00	Yes	41	LRP	OM
62	F	31	Extremity	Soft tissue sarcoma	1	No	0	N/A	0.00	Yes	18	LRP	OM
64	F	55	Colon	Adenocarcinoma	2	No	0	N/A	0.00	Yes	29	LRP	OM
68	F	81	Head and neck	Adenoid cystic carcinoma	1	No	1	12	0.05	Yes	21	LRP	OM
71	F	66	Extremity	Soft tissue sarcoma	3	No	1	8	0.03	Yes	38	LRP	OM
75	F	60	Extremity	Soft tissue sarcoma	1	No	1	74	0.01	No	120	LRP	OM
76	F	62	Head and neck	Squamous cell carcinoma	1	No	0	N/A	0.00	No	20	LRP	OM
77	M	56	Colon	Adenocarcinoma	1	No	0	N/A	0.00	Yes	81	LRP	OM
83	M	51	Extremity	Soft tissue sarcoma	3	No	2	42	0.02	Yes	121	LRP	OM
87	M	62	Thyroid	Adenocarcinoma	1	No	3	22	0.04	Yes	69	LRP	OM

2	F	52	Colon	Adenocarcinoma	2	No	10	4	0.37	No	27	HRP	PM
3	F	58	Colon	Adenocarcinoma	2	Yes	7	3	0.70	No	10	HRP	PM
5	F	67	Extremity	Osteosarcoma	1	No	10	8	0.83	No	12	HRP	PM
7	F	41	Anus	Squamous cell carcinoma	5	Yes	10	7	0.59	Yes	17	HRP	PM
25	F	62	Renal	Renal cell carcinoma	5	No	10	6	0.59	No	17	HRP	PM
26	M	56	Bladder	Transitional cell carcinoma	1	No	10	5	0.42	Yes	24	HRP	PM
28	M	56	Renal	Renal cell carcinoma	2	No	10	8	0.59	No	17	HRP	PM
29	F	54	Salivary gland	Adenoid cystic carcinoma	1	Yes	10	3	2.00	No	5	HRP	PM
34	M	61	Extremity	Soft tissue sarcoma	1	No	10	9	0.37	No	27	HRP	PM
46	M	62	Renal	Renal cell carcinoma	1	No	10	6	0.37	No	27	HRP	PM
60	F	65	Chest wall	Soft tissue sarcoma	1	No	10	7	0.53	No	19	HRP	PM
69	M	48	Colon	Adenocarcinoma	2	No	10	2	0.33	No	30	HRP	PM
73	M	52	Head and neck	Squamous cell carcinoma	3	No	10	9	0.50	No	20	HRP	PM
78	M	66	Bile duct	Cholangiocarcinoma	2	Yes	10	11	0.50	Yes	20	HRP	PM
82	M	83	Head and neck	Angiosarcoma	2	Yes	10	3	0.77	No	13	HRP	PM
90	M	68	Esophagus	Adenosquamous	1	No	7	2	1.40	No	5	HRP	PM
13	F	38	Extremity	Soft tissue sarcoma	1	No	3	10	0.09	Yes	32	IRP	OM
16	M	24	Extremity	Osteosarcoma	1	No	8	16	0.13	No	61	IRP	OM
17	F	70	Colon	Adenocarcinoma	1	No	10	13	0.16	No	62	IRP	OM
33	F	46	Renal	Renal cell carcinoma	1	No	9	9	0.16	Yes	55	IRP	OM
56	M	68	Lung	Soft tissue sarcoma	2	No	5	5	0.24	No	21	IRP	OM
63	M	45	Extremity	Soft tissue sarcoma	2	No	10	19	0.13	No	78	IRP	OM
81	M	72	Extremity	Soft tissue sarcoma	1	No	10	6	0.28	No	36	IRP	OM
1	M	68	Renal	Renal cell carcinoma	1	No	10	8	0.21	No	47	IRP	PM
9	M	73	Colon	Adenocarcinoma	1	No	10	21	0.14	No	70	IRP	PM
37	F	29	Sacrum	Soft tissue sarcoma	1	No	10	20	0.14	No	74	IRP	PM
44	M	73	Colon	Adenocarcinoma	4	No	7	10	0.13	No	52	IRP	PM
58	M	32	Shoulder	Soft tissue sarcoma	1	Yes	10	18	0.20	No	50	IRP	PM
67	M	55	Renal	Renal cell carcinoma	4	No	10	6	0.26	No	38	IRP	PM
79	M	71	Head and neck	Melanoma	1	No	10	43	0.30	Yes	43	IRP	PM
88	M	63	Renal	Renal cell carcinoma	2	No	10	13	0.20	No	50	IRP	PM
