# Supplementary material for: Oligo- and Polymetastatic Progression in Lung Metastasis(es) Patients Is Associated with Specific MicroRNAs
Source: PLoS One. 2012 Dec 10;7(12):e50141. doi: 10.1371/journal.pone.0050141 (PMC3518475; doi:10.1371/journal.pone.0050141)
Supplement: Tables S2 — Characteristics of patients with LRP and HRP progression (S2A) and oligometastatic (OM) and polymetastatic (PM) progression (S2B) in lung metastasis samples. Legend: two-tailed Fisher's Exact Test (FET); two-tailed non-parametric Mann Whitney Test (MWT); log-rank Mantel-Cox test (Log-rank); * = statistically significant. (PDF) [file pone.0050141.s005.pdf]

Supplementary Tables S2A and S2B. Characteristics of patients with LRP and HRP progression (S2A) and oligometastatic (OM) and polymetastatic (PM) progression (S2B) in lung metastasis samples. Legend: Student's t-test (t-test); two-tailed Fisher's Exact Test (FET); two-tailed non-parametric Mann Whitney Test (MWT); log-rank Mantel-Cox test (Log-rank); *=statistically significant; *ns* = not significant

Supplementary Table S2A

**Patients** (#)	**Overall Group** (N=63)	**Cumulative LRP and HRP** (N=48)	**LRP progression** (N=32)	**HRP progression** (N=16)	**p-value**
**Median age at surgery** (Range)	58 (24-83)	58 (26-83)	58 (26-82)	59 (41-83)	0.67 *ns* (t-test)
**Sex**					
Female (%)	32	28	21 (75%)	7 (25%)	0.22 *ns* (FET)
Male (%)	31	20	11 (55%)	9 (45%)	
**Primary tumor type**					
Gastrointestinal	16	13	8	5	0.74 *ns* (FET)
Musculoskeletal/Soft Tissue	18	12	9	3	0.73 *ns* (FET)
Genitourinary	11	7	3	4	0.20 *ns* (FET)
Head and Neck	8	7	4	3	0.67 *ns* (FET)
Breast	4	4	4	0	0.29 *ns* (FET)
Gynecologic	3	3	3	0	0.54 *ns* (FET)
Hepatobiliary	2	2	1	1	1.0 *ns* (FET)
Lung	1	0	0	0	1.0 *ns* (FET)
**Histology**					
Adenocarcinoma	23	19	16	3	0.06 *ns* (FET)
Sarcoma	22	15	11	4	0.74 *ns* (FET)
Renal Cell Carcinoma	7	4	1	3	0.10 *ns* (FET)
Squamous Cell Carcinoma	3	3	1	2	0.25 *ns* (FET)
Adenoid Cystic Carcinoma	3	3	2	1	1.0 *ns* (FET)
Other	5	4	1	3	
**Median # metastasis(es) at surgery** (Range)	1 (1-5)	1 (1-5)	1 (1-3)	2 (1-5)	0.15 *ns* (MWT)
**Median # of total recurrent metastasis(es) following surgery** (Range)	3 (0-10)	1 (0-10)	0 (0-3)	10 (7-10)	**<0.0001*** (MWT)
**Survival** (total # alive throughout follow-up/total per group)	31/63	28/48	25/32	3/16	**0.0001*** (FET)
**Median follow-up time (months)** (Range)	47 (5-149)	38.5 (5-149)	63.5 (18-149)	18 (5-24)	**<0.0001*** (Log-rank)

Supplementary Table S2B

**Patients** (#)	**All** (N=63)	**OM progression** (N=39)	**PM progression** (N=24)	**p-value**
**Median age at surgery** (Range)	58 (24-83)	58 (24-82)	61 (29-83)	0.54 *ns* (t-test)
**Sex**				
Female (%)	32	24 (62%)	8 (33%)	0.04* (FET)
Male (%)	31	15 (38%)	16 (67%)	
**Primary tumor type**				
Gastrointestinal	16	9	7	0.77 *ns* (FET)
Musculoskeletal/Soft Tissue	18	13	5	0.39 *ns* (FET)
Genitourinary	11	4	7	0.09 *ns* (FET)
Head and Neck	8	4	4	0.47 *ns* (FET)
Breast	4	4	0	0.29 *ns* (FET)
Gynecologic	3	3	0	0.28 *ns* (FET)
Hepatobiliary	2	1	1	1.0 *ns* (FET)
Lung	1	1	0	1.0 *ns* (FET)
**Histology**				
Adenocarcinoma	22	17	5	0.10 *ns* (FET)
Sarcoma	22	16	6	0.28 *ns* (FET)
Renal Cell Carcinoma	8	2	6	0.05* (FET)
Squamous Cell Carcinoma	3	1	2	0.55 *ns* (FET)
Adenoid Cystic Carcinoma	3	2	1	1.0 *ns* (FET)
Other	5	1	4	
**Median # metastasis(es) at surgery** (Range)	1 (1-5)	1 (1-3)	1.5 (1-5)	0.14 *ns* (MWT)
**Median # of total recurrent metastasis(es) following surgery** (Range)	3 (0-10)	0 (0-10)	10 (7-10)	**<0.0001*** (MWT)
**Survival** (total # alive throughout follow-up/total per group)	31/63	27/39	4/24	**7.02E-5*** (FET)
**Median follow-up time (months)** (Range)	47 (5-149)	59 (18-149)	25.5 (5-74)	**<0.0001*** (Log-rank)
