# Supplementary material for: Oligo- and Polymetastatic Progression in Lung Metastasis(es) Patients Is Associated with Specific MicroRNAs
Source: PLoS One. 2012 Dec 10;7(12):e50141. doi: 10.1371/journal.pone.0050141 (PMC3518475; doi:10.1371/journal.pone.0050141)
Supplement: Table S3 — Classification of primary and metastatic patient samples of the independent validation dataset into stratified metastatic phenotypes. For each patient in the independent validation study [GEO: GSE25552], the following clinical information is provided and organized by metastatic rate phenotype: Patient ID; Primary vs. metastasis tumor type; Time to first metastatic recurrence following radiotherapy (months); Rate of recurrent metastasis(es) per month following radiotherapy throughout follow-up, Alive status (Yes/No); Survival (months); Metastatic rate phenotype (HRP, LRP, IRP); Oligo- vs. poly-metastatic progression (OM, PM). Note for this study that all HRP patients must also be classified as PM and all LRP patients must be classified as OM. The * in the Survival (months) column represents updated follow-up since the original publication [22]. (PDF) [file pone.0050141.s006.pdf]

Supplementary Table S3. Classification of primary and metastatic patient samples of the independent validation dataset into stratified metastatic phenotypes. For each patient in the independent validation study [GEO: GSE25552], the following clinical information is provided and organized by metastatic rate phenotype: Patient ID; Primary vs. metastasis tumor type; Time to first metastatic recurrence following radiotherapy (months); Rate of recurrent metastasis(es) per month following radiotherapy throughout follow-up, Alive status (Yes/No); Survival (months); Metastatic rate phenotype: HRP, LRP, IRP; Oligo- vs. poly-metastatic progression: OM, PM. Note for this study that all HRP patients must also be classified as PM and all LRP patients must be classified as OM. The * in the Survival (months) column represents updated follow-up since the original publication [Lussier et al 2011].

Patient ID	Primary vs. metastasis tumor type	Time to first metastatic recurrence following radiotherapy (months)	Rate of recurrent metastasis(es) per month following radiotherapy	Alive	Survival (months)	Metastatic rate phenotype	Oligo- vs.poly-metastatic progression
1a	Primary	4.0	1.00	No	10.5	HRP	PM
204b	Metastasis	17.0	0.55	No	18.2	HRP	PM
230a	Primary	1.0	0.91	No	11.1	HRP	PM
236b	Metastasis	17.0	0.38	Yes	*23.5	HRP	PM
2a	Primary	23.0	0.36	No	27.4	HRP	PM
39a	Primary	1.0	0.83	No	11.9	HRP	PM
46a	Primary	1.0	1.25	No	8.4	HRP	PM
4a	Primary	6.0	0.34	No	29.5	HRP	PM
4b	Metastasis	6.0	0.34	No	29.5	HRP	PM
59b	Metastasis	1.0	0.56	No	17.9	HRP	PM
6a	Primary	4.0	0.83	No	11.5	HRP	PM
6b1	Metastasis	4.0	0.87	No	11.5	HRP	PM
6b2	Metastasis	4.0	0.87	No	11.5	HRP	PM
7b	Metastasis	1.0	0.79	No	12.6	HRP	PM
18a	Primary	11.0	0.00	No	10.2	LRP	OM
18b	Metastasis	10.2	0.00	No	10.2	LRP	OM
217a	Primary	29.0	0.00	Yes	32.9	LRP	OM
221a	Primary	35.0	0.00	Yes	35.8	LRP	OM
228a	Primary	7.0	0.00	No	14.0	LRP	OM
231a	Primary	22.0	0.00	Yes	22.0	LRP	OM
27a	Primary	54.0	0.00	Yes	59.8	LRP	OM
31b	Metastasis	31.0	0.03	Yes	*57.4	LRP	OM
52a	Primary	39.0	0.00	Yes	40.8	LRP	OM
57a	Primary	26.0	0.00	Yes	26.2	LRP	OM
13a	Primary	1.0	0.33	No	14.9	IRP	OM
15a	Primary	4.0	0.12	Yes	33.6	IRP	OM
15b	Metastasis	4.0	0.12	Yes	33.6	IRP	OM
202b	Metastasis	21.0	0.09	Yes	*55.4	IRP	OM
209a	Primary	13.0	0.18	No	17.4	IRP	OM
220a	Primary	2.0	0.11	Yes	34.4	IRP	OM
22a	Primary	1.0	0.10	Yes	29.2	IRP	OM
22b	Metastasis	1.0	0.10	Yes	29.2	IRP	OM
23b	Metastasis	12.0	0.20	Yes	*50.5	IRP	OM
244a	Primary	3.0	0.25	Yes	12.7	IRP	OM
26a	Primary	2.0	0.08	Yes	58.7	IRP	OM
49b1	Metastasis	12.0	0.16	Yes	*44.6	IRP	OM
49b2	Metastasis	12.0	0.16	Yes	*44.6	IRP	OM
58a	Primary	1.0	0.29	Yes	34.5	IRP	OM
5b	Metastasis	1.0	0.11	No	18.2	IRP	OM
60a	Primary	34.0	0.14	Yes	37.5	IRP	OM
65a	Primary	22.0	0.15	Yes	32.6	IRP	OM
