# Supplementary material for: Oligo- and Polymetastatic Progression in Lung Metastasis(es) Patients Is Associated with Specific MicroRNAs
Source: PLoS One. 2012 Dec 10;7(12):e50141. doi: 10.1371/journal.pone.0050141 (PMC3518475; doi:10.1371/journal.pone.0050141)
Supplement: Table S4 — Evidence of tumor suppression and tumor promotion functions of prioritized microRNAs between LRP and HRP patients of the lung metastasis dataset. The tumor suppressing and tumor promoting functions of each of the 40 prioritized microRNAs between LRP and HRP patients of the lung metastasis dataset (Table 1) were investigated. Inclusion criteria used for citations required that each study provide experimental evidence for the role of the microRNA in a cancer context (i.e. cancer cell culture model, animal model of cancer, or human cancer samples) and that functional assays were performed. For instance, differential expression of a microRNA between cancer and control states were not considered as experimental evidence if they were not accompanied with experiments examining the functional role in either suppressing or promoting the cancer phenotype in regards to the expression results. Experiments conducted in human samples were considered if expression results were correlated to meaningful clinical variables (e.g. survival outcome, metastatic progression). (PDF) [file pone.0050141.s007.pdf]

Supplementary Table S4. Evidence of tumor suppression and tumor promotion functions of prioritized microRNAs betweenLRP and HRP patients of the lung metastasis dataset. The tumor suppressing and tumor promoting functions of each of the 40 prioritized microRNAs between LRP and HRP patients of the lung metastasis dataset (**Table 1**) were investigated. Inclusion criteria used for citations required that each study provide experimental evidence for the role of the microRNA in a cancer context (i.e. cancer cell culture model, animal model of cancer, or human cancer samples) and that functional assays were performed. For instance, differential expression of a microRNA between cancer and control states were not considered as experimental evidence if they were not accompanied with experiments examining the functional role in either suppressing or promoting the cancer phenotype in regards to the expression results. Experiments conducted in human samples were considered if expression results were correlated to meaningful clinical variables (e.g. survival outcome, metastatic progression).

miRNA	Evidence of tumor suppression function				Evidence of tumor promotion function			
	PMID	Author	Year	Journal	PMID	Author	Year	Journal
let-7b	21087605	Fu TY et al	2011	Exp Cell Res	-	-	-	-
let-7c	21984339	Han HB et al	2012	J Pathol	-	-	-	-
miR-127-3p	-	-	-	-	-	-	-	-
miR-127-5p	-	-	-	-	-	-	-	-
miR-128	18810376;<br>19010882;<br>19713529	Zhang Y et al;<br>Godlweski J et al;<br>Evangelistic C et al	2009;<br>2008;<br>2009	J Mol Med (Berl); Cancer Res;<br>FASEB J	-	-	-	-
miR-133a	22266319;<br>22292984	Kinoshita T et al;<br>Wu ZS et al	2012;<br>2012	Biochem Biophys Res Commun;<br>BMC Cancer	-	-	-	-
miR-135a	22439757;<br>21888875	Chen Y et al;<br>Liu S et al	2012;<br>2012	BMC Cancer;<br>J Hepatol				
miR-153	19676043	Xu J et al	2010	Int J Cancer	20028871	Myatt SS et al	2010	Cancer Res
miR-154	21128228	Wang W et al	2011	Int J Cancer	-	-	-	-
miR-191	-	-	-	-	21880514	Leite KR et al	2011	Urol Oncol
miR-199a-5p	22438992;<br>20799954	Huang L et al;<br>Shen Q et al	2012;<br>2010	PLoS One;<br>Mol Cancer	-	-	-	-
miR-199b-5p	19308264	Garzia L et al	2009	PLoS One				
miR-205	19665999;<br>19238171	Song H et al;<br>Wu H et al	2009;<br>2009	Biochem Biophys Res Commun;<br>Cell Res	20103531	Greene SB et al	2010	J Cell Sci
miR-296-3p	-	-	-	-	-	-	-	-
miR-298	-	-	-	-	-	-	-	-
miR-299-3p	-	-	-	-	-	-	-	-
miR-328	20211135	Eiring AM et al	2010	Cell	21448905	Arora S et al	2011	Int J Cancer
miR-329	-	-	-	-	-	-	-	-
miR-330-5p	19597470	Lee KH et al	2009	Oncogene	-	-	-	-
miR-369-3p	-	-	-	-	-	-	-	-
miR-380	-	-	-	-	20871609	Swarbrick A et al	2010	Nat Med
miR-412	-	-	-	-	-	-	-	-
miR-448	-	-	-	-	-	-	-	-
miR-453	-	-	-	-	-	-	-	-
miR-485-3p	-	-	-	-	-	-	-	-
miR-485-5p	21083603	Kim TH et al	2010	Histopathology	-	-	-	-
miR-491-5p	21831363	Yan W et al	2011	Brain Res	-	-	-	-
miR-502-5p	-	-	-	-	-	-	-	-
miR-506	21726609	Zhao Y et al	2011	Toxicol Lett				
miR-520a-3p	-	-	-	-	20190813	Wu S et al	2010	Oncogene
miR-520g	-	-	-	-	-	-	-	-
miR-541	-	-	-	-	-	-	-	-
miR-544	22037351	Thayanithy V et al	2012	Bone	-	-	-	-
miR-576-5p	-	-	-	-	-	-	-	-
miR-582-5p								
miR-654-5p	21343391	Ostling P et al	2011	Cancer Res	-	-	-	-
miR-655	-	-	-	-	-	-	-	-
miR-672	-	-	-	-	-	-	-	-
miR-887	-	-	-	-	-	-	-	-
miR-98	22492871;<br>20525879	Panda H et al;<br>Wang YC et al	2012;<br>2010	J Clin Endocrinol Metab;<br>Carcinogenesis	-	-	-	-
